# Required Art-Based Curriculum Improves Essential Qualities in Medical Students: A Three-Year Study With In-Person and Remote Art Observation Experiences

**DOI:** 10.7759/cureus.82022

**Published:** 2025-04-10

**Authors:** Schoen W Kruse, Sarah E Getch, Anshul Kumar, Marissa Roffler

**Affiliations:** 1 Academic Affairs, Texas Tech University Health Sciences Center, Lubbock, USA; 2 Clinical Psychology, Kansas City University, Kansas City, USA; 3 Health Professions Education, Massachusetts General Hospital (MGH) Institute of Health Professions, Boston, USA; 4 Psychology, Rockhurst University, Kansas City, USA

**Keywords:** ambiguity, art in medicine, museum, online medical education, remote education

## Abstract

Medical education is increasingly challenged to develop a curriculum that addresses ambiguity, empathy, and perspective-taking. We developed a required art-based curriculum for all first-year medical students to address this need. Within this course, students use art observation and the Visual Thinking Strategies (VTS) question framework to explore interprofessional collaborative practice, ambiguity in healthcare, and perspective-taking. The purpose of this study was to examine if the art-based curriculum was associated with changes in tolerance for ambiguity, perspective-taking, and empathy in medical students. Changes in these qualities were evaluated between in-person, emergency remote, and planned remote sessions.

From September 2018 to May 2021, 794 first-year medical students from two campuses participated in a two-hour art observation session where VTS was used to prompt discussion. All participants were surveyed in pre- and post-art observation activities in tolerance for ambiguity, empathy, and perspective-taking. A linear mixed-effects regression model was used to measure pre/post changes for each learning modality (in-person, emergency remote, planned remote).

The two-hour art observation activity using VTS was associated with an increase in tolerance for ambiguity of 0.19 (95% CI: 0.15 to 0.23) average TFA scale points. We observed no difference in outcomes across academic years, museums, emergency remote, or planned remote experiences. We did not detect significant differences in either perspective-taking or empathy.

We have developed a curriculum that improves qualities that are essential for practice in complex and evolving health systems. These improvements are observed independent of learning modality (in an art museum, emergency remote teaching, or planned remote teaching), creating an opportunity to engage with students and programs across the spectrum of health professions education, independent of location. Providing access to educational programming that addresses essential qualities and behaviors of healthcare providers has the potential to improve patient outcomes through team-based healthcare and interprofessional collaborative practice.

## Introduction

Diagnostic skill development is an essential, and historically central, driving force of medical education. Indeed, educators in health professions have a responsibility to identify and implement curricula that develop diagnostic and procedural skills so that learners are prepared to enter postgraduate training programs. Empirical research collected over the past two decades suggests that several personality attributes, including empathy, are among significant predictors of clinical competence of physicians-in-training [1,2] and of positive patient outcomes [3,4]. Empathy has been described as one major element of professionalism in medicine [5] and the most frequently mentioned personality attribute of the humanistic physician [6,7]. As such, health professions programs must also develop curricula that teach and cultivate these positive personality attributes that will result in improved healthcare delivery and patient outcomes.

Another facet of clinical competency includes being comfortable with and directly addressing uncertainty. Tolerance for ambiguity (TFA) enables health professionals to navigate situations in which there is incomplete information or multiple answers. Ambiguity intolerance has been associated with traits such as authoritarianism, dogmatism, and ethnic prejudice, which contradict patient-centered qualities underlying ethical medical practice. Low tolerance for ambiguity has been associated with increased test-ordering tendencies and failure to comply with evidence-based guidelines, defensive practice, and discomfort in the context of death and grief [8]. Empathy is inherently related not only to TFA but also to communication, given that better communication can impact the extent to which an individual is able to participate in empathetic perspective-taking.

Perspective-taking, a process in which one person strives to realize what another is thinking and feeling, has been shown to enhance empathy in both non-medical and medical contexts. Engaging in the perspective-taking process improves communication, facilitates altruism, and decreases stereotyping and prejudice [9]. Perspective-taking is an essential activity of interprofessional education and collaborative practice and is a critical skill for all healthcare professionals. 

Over the past 30 years, health professions programs have increasingly included the humanities in the curriculum to address the need to develop positive personality attributes in healthcare providers [10,11]. Instruction in the humanities has mostly been used to build observation skills, but also reflection, introspection, tolerance for ambiguity, and flexible thinking [10]. Recent reviews of visual art instruction in medical education identified common themes among institutions that have incorporated humanities in medical education [11,12]). Across the health professions, common goals of humanities within medical education include the development of observation skills, diagnostic skills, empathy, team building, communication skills, resilience, and cultural sensitivity. Most humanities curricula in medical education occur in the preclinical years and use validated pedagogy such as Visual Thinking Strategies or Artful Thinking. 

There are nearly 70 medical schools in the USA, Canada, Australia, and Italy that offer courses in the arts for their students. The vast majority of these programs offer these courses as electives, with less than 10 courses existing as required curricular elements within their respective programs [11]. Although many health professions programs do include humanities within the curriculum, there is little rigorous published data that investigates the extent to which art-based humanities curricula develop and cultivate positive personality attributes. While there is strong evidence that learners enjoy and are satisfied with arts-based coursework, only a limited number of studies show that art-based humanities courses promote empathy, team building, communication skills, wellness, resilience, and cultural sensitivity in health professions students [11]. Published reports do show that visual arts-based education can promote clinical excellence by enhancing communication and interpersonal skills, professionalism and humanism, diagnostic acumen, and clinical reasoning [13]. However, large-scale initiatives across multiple sites and multiple class cohorts that show consistent positive outcomes are lacking. In order to better understand the role of visual arts instruction in health professions education, more robust, evidence-based approaches with many participants are needed.

At Kansas City University (KCU) College of Osteopathic Medicine (COM), the Art, Observation, and Medicine (ArtMed) course uses visual arts to teach diagnostic skill development, enhance communication skills, and emphasize the importance of empathy and relationship building in health professions students. ArtMed is a required course that was designed by author S.K. for year one osteopathic medical students (DO) at both the KCU-Kansas City campus and KCU-Joplin campus and aims to help forge connections between active art examination and diagnosis, awareness of tolerance for ambiguity in healthcare, teamwork, perspective-taking, communication skills, and empathy. The course is taught through partnerships between KCU, the Kemper Museum of Contemporary Art (Kansas City, MO), and the Crystal Bridges Museum of American Art (Bentonville, AR) [14].

The purpose of this study is to investigate changes in tolerance for ambiguity, perspective-taking, and empathy in medical students associated with their participation in the two-hour art observation activity in the ArtMed course. We hypothesized that students would demonstrate a statistically significant increase in tolerance for ambiguity, perspective-taking, and empathy following the ArtMed course. This study included student participants across three academic years of instruction at two campuses. When the COM had to swiftly transition to emergency remote teaching in the spring of 2020, the art observation activity followed suit and transitioned to an online platform (emergency remote teaching in this study). The program was fully online in 2020-2021 (planned remote teaching in this study), providing an opportunity to evaluate any associated changes in tolerance for ambiguity, perspective-taking, and empathy due to in-person, emergency remote, and planned remote art observation encounters. This article was previously presented as a short communication at the 2023 AMEE Annual Meeting on August 29, 2023.

## Materials and methods

This study evaluated a required component of the KCU College of Osteopathic Medicine (COM) curriculum. The course, Art Observation and Medicine (ArtMed), uses Visual Thinking Strategies (VTS) during art observation in an art museum or online (Zoom virtual classroom) setting. The purpose of the course is to increase empathy, tolerance for ambiguity, and perspective-taking in medical students to promote mindful observation and prepare students to interact with patients. A total of 794 first-year COM students enrolled in the ArtMed course and participated in the study between September 2018 and May 2021. 

Students participated in one of two museum-based ArtMed activities. Kansas City-COM students attended the Kemper Museum of Contemporary Art in Kansas City, MO, and Joplin-COM students attended the Crystal Bridges Museum of American Art in Bentonville, AR. COM students enrolled in the ArtMed course in AY19-20 were categorized as emergency remote if their art observation session took place after March 2020, when KCU abruptly transitioned all education to emergency remote teaching due to the worldwide COVID-19 pandemic. All student participants experienced the art museum in a planned remote capacity in AY20-21, as the entire course remained a remote experience for the duration of the academic year. Fewer participants were recruited for the AY20-21 due to unanticipated interruptions in the research study as a result of the COVID-19 pandemic.

Participants completed surveys to measure three dependent variable constructs: Tolerance for Ambiguity (TFA) [15], perspective-taking, and empathy, which were measured using the Interpersonal Reactivity Index (IRI) with subscales measuring empathic concern and perspective-taking [16-18]. Higher survey scores indicate higher levels of each construct. All participants completed measurement pre-tests within one week prior to the museum activity and completed the post-tests within one week after the museum activity (remote or in person). Participants were also asked to complete several questions to account for possible covariates. Demographic questions, including gender and specialty interest, and the date of the museum session were recorded. All survey responses were completed using an online survey platform. In AY18-19 and AY19-20, completion of TFA and IRI were course requirements. During those academic years, students were given the opportunity to opt out of having their data used for research purposes. An online survey link was provided to have students indicate if they did not want their data to be used. This link was monitored and available only to a third-party staff member. In AY20-21, students were given the opportunity to opt-in to the study. All data was de-identified by the third-party staff member before it was delivered to the research team. All student communication, including links to pre- and post-tests and opt-out forms, occurred in the learning management system (Blackboard, Canvas). Pre- and post-test responses were collected in Blackboard, Canvas, and Qualtrics.

**Figure 1:**
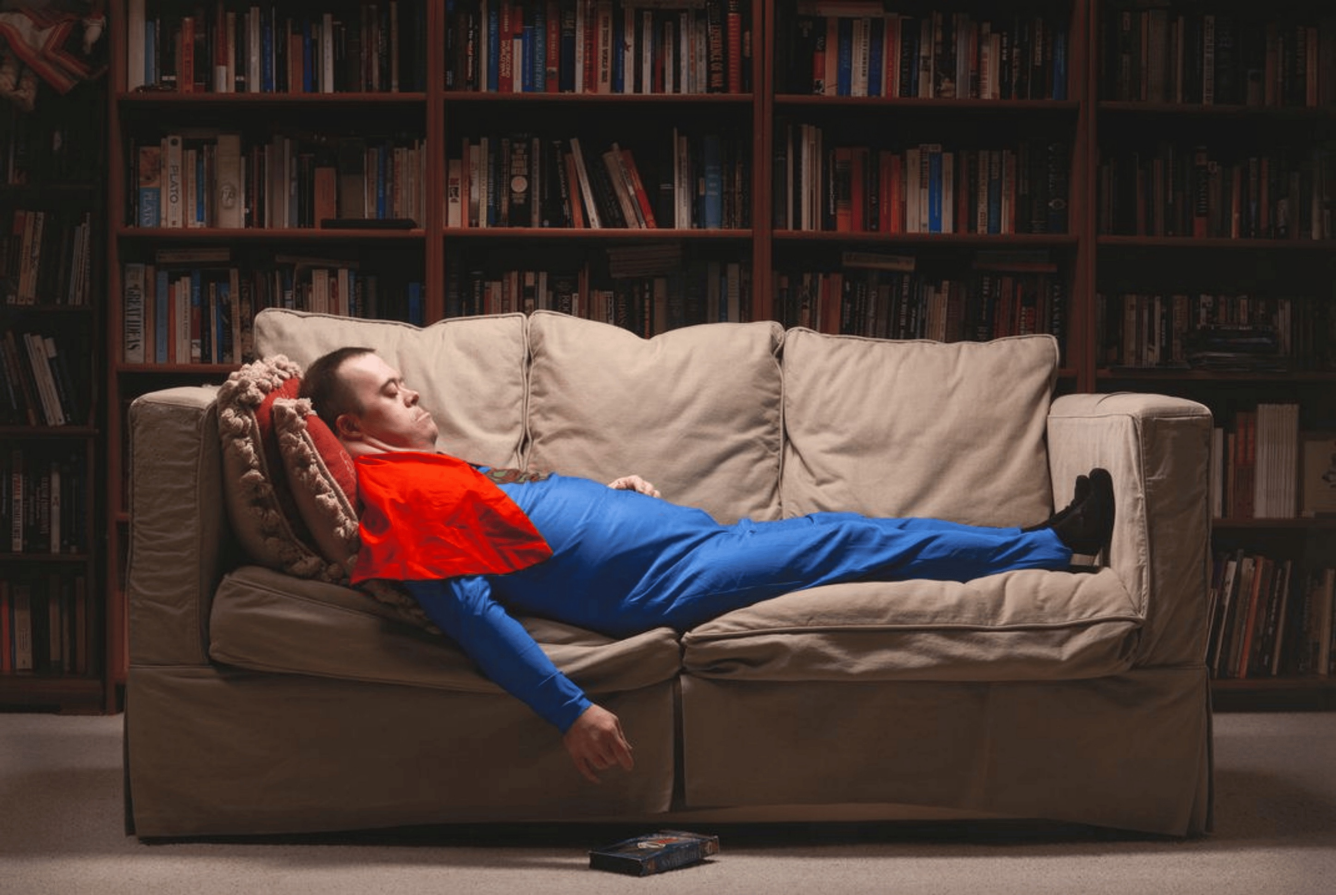
Example image used in ArtMed VTS experience Michael #145973 by Rick Ashley. This photo was a finalist in the Outwin Boochever Portrait Competition in 2016. This triannual competition is supported by the Smithsonian National Portrait Gallery and travels nationally and internationally. The Kemper Museum of Contemporary Art hosted the traveling gallery of finalists in the fall of 2017. Reprinted with permission. Source: https://portraitcompetition.si.edu/exhibition/2016-outwin-boochever-portrait-competition/michael-145973/ [14].

Students viewed approximately five pieces of art in the two-hour activity using VTS (see example image in Figure 1). The artwork was chosen by museum educators for the purpose of rich and robust discussion using the VTS question framework and was not medical in nature. The VTS prompts are as follows: "What is going on in this picture? What do you see that makes you think that? What more can we find?"[19]. Museum docents engaged in reflective listening and repeated observations made by students while pointing out the elements in the piece of art. Students were asked to make additional observations or discuss if they agree or disagree with other observations. At the conclusion of the direct observation of art images, structured debriefing questions were asked of each group of participants. Questions focused each participant’s attention on topics such as ambiguity in patient care, team-based healthcare, interprofessional collaborative practice, and perspective-taking. Example debriefing questions are listed below.

A) What did you notice about your observations? Your peer’s observations? B) Did you ever change your mind about your initial impressions? C) What did you notice about yourself when you encountered something that was confusing, vague, or ambiguous?

Outcomes of interest (TFA, perspective-taking, and empathy) and the post-pre delta were calculated separately for each of the three different learning modalities over the three-year study period. A linear mixed effects regression model (LMER) was used to control for nesting of pre- and post-data within participants and participants within museum visit groups. The rationale for utilizing a nested model was based on the possible non-independence of student scores for those students who participated in the same group. Our data set was arranged in a long format, with each row representing a single measurement (pre or post) of each student, resulting in a data set with 1588 rows in total (two per participant). Independent variables were the interaction of pre-post dummy variables and learning modality dummy variables, pre-post academic year dummy variables, gender dummy variables, and student age. All diagnostic tests of the regression model were performed and passed. Statistical analysis was conducted using the lmer function within the lme4 package [20] in R version 3.6.3 (R Foundation for Statistical Computing, Vienna, Austria). We ran one regression model for each outcome of interest, with a random effect at the participant level. Additional details of the results are available upon request.

## Results

A breakdown of the student participants is shown in Table 1 and highlights the multi-year study, multiple museums for in-person experiences, and three distinct learning modalities for the art museum experience in the ArtMed course. Demographic data (identified gender, age) of study participants as a function of art museum experience are shown in Tables 2, 3.

**Table 1:** Number of year one medical student participants by academic year and art museum experience Percentage of 794 total student participants in parenthesis ^$^In AY 18-19, there were 263 student participants at the Kemper Museum of Contemporary Art and 159 student participants at Crystal Bridges Museum of American Art (62% and 38%, respectively, of the 422 in-person participants). ^*^In AY 19-20, there were 163 student participants at the Kemper Museum of Contemporary Art and 87 student participants at Crystal Bridges Museum of American Art (65% and 35%, respectively, of the 250 in-person participants).

Art museum experience	AY18-19	AY19-20	AY20-21	Total
In-person	422^$^ (53)	250^*^ (31)	0 (0)	672 (85)
Emergency remote	0 (0)	90 (11)	0 (0)	90 (11)
Planned remote	0 (0)	0 (0)	32 (4)	32 (4)
Total	422 (53)	340 (43)	32 (4)	794

**Table 2:** Identified gender of study participants Percentage of 794 total student participants in parenthesis

Identified Gender	In-person	Emergency remote	Planned remote	Total
Male	354 (45)	53 (7)	12 (2)	419 (53)
Female	314 (40)	37 (5)	20 (3)	371 (47)
NA	4 (1)	0 (0)	0 (0)	4 (1)

**Table 3:** Identified age of study participants Percentage of 794 total student participants in parenthesis Two in-person and one planned remote participant did not report an age

Age	In-person	Emergency remote	Planned remote	Total
≤21	15 (2)	0 (0)	0 (0)	15 (2)
22-23	222 (28)	16 (2)	5 (1)	243 (31)
24-25	254 (32)	44 (6)	16 (2)	314 (40)
26-27	97 (12)	19 (2)	6 (1)	122 (15)
28-29	40 (5)	6 (1)	3 (1)	49 (6)
≥30	42 (5)	5 (1)	1 (1)	48 (6)
Mean Age	24.8	25.1	25.2	25.1

There were 794 participants in the study across three years. A total of 672 (85%) of those 794 were in the museum in-person, 90 (11%) for emergency remote (unexpected switch to remote learning at the start of the COVID-19 pandemic), and 32 (4%) for planned remote. Results from our LMER model (Table 4, 5) show an average pre-post increase in TFA score of 0.19 points (95% CI: 0.15-0.23) for in-person learners. Interaction terms for emergency remote and planned remote learners suggest possible additional average increases (beyond that of in-person learners) of 0.05 points (95% CI: -0.07-0.17) and 0.15 points (95% CI: -0.05-0.36), respectively. More information about these results is available upon request.

**Table 4:** Pre- and post-museum outcomes for tolerance for ambiguity, perspective-taking, and empathic concern measurements Standard deviations in parentheses. ^*^Indicates a statistically significant difference ^^^Adjusted for controls using LMER model N = 794 across 2 campuses and 2 museums, including emergency remote and planned remote learning experiences

Measure	Pre-test	Post-test	Delta
Tolerance for Ambiguity (scale 1-6)	3.20 (0.78)	3.40 (0.87)	0.19^*^^
Perspective-Taking (scale 0-4)	2.39 (0.41)	2.43 (0.41)	0.04
Empathetic Concern (scale 0-4)	2.12 (0.34)	2.11 (0.34)	0.01

**Table 5:** LMER model results (dependent variable: average tolerance for ambiguity) A total of 785 participants with complete data included in regression model Note: *p<0.1 **p<0.05 ***p<0.01

Independent Variable	Estimate	SE	95% CI	Variable Details
Pre/Post Change	0.19***	.02	(0.15, 0.23)	Time variable: 1= post ArtMed, 0= pre ArtMed
Gender	-0.06	0.06	(-0.17, 0.05)	1= female, 0= male
Age	0.03***	0.01	(0.01, 0.05)	Years, (min= 20, max= 40)
Emergency Remote	-0.10	0.10	(-0.29, 0.09)	1= emergency remote, 0= in person
Planned Remote	-0.05	0.16	(-0.37, 0.27)	1= planned remote, 0= in person
Emergency Remote * Post	0.05	0.06	(-0.07, 0.17)	interaction term
Planned Remote * Post	0.15	0.10	(-0.05, 0.36)	interaction term
Intercept	2.45***	0.27	(1.93, 2.97)	
Pseudo-R² (total)	0.79			

We found the average pre-test mean increased by 0.04 to the post-test mean score in the IRI perspective-taking subscale. We observed a 0.01 decrease in the IRI empathy subscale mean score from the pre-test to the post-test (Table 4). 

## Discussion

KCU’s visual arts-based activity using Visual Thinking Strategies is part of a growing movement to integrate the arts and humanities across medical and health professions education. While many programs use the arts and humanities to improve clinically relevant skills such as observation and critical thinking [13], visual art observation and discussion can also provide an opportunity to enhance communication skills and build tolerance for ambiguity, empathy, and perspective-taking. By developing a curriculum to enhance communication and interpersonal skills, humanism, and professionalism, medical and other health professions programs better prepare future physicians and health practitioners to address the healthcare and health system challenges of society. The ArtMed course using VTS as a questioning framework at KCU addresses these challenges and is associated with changes in essential qualities in our medical students.

We observed a significant average improvement in tolerance for ambiguity based on the two-hour museum session in the ArtMed course at KCU. The observed improvement in tolerance for ambiguity was consistent across multiple different environments. We observed consistent increases in the TFA score for students participating in a museum setting at two different museum locations with different museum educators. In addition, we observed consistent increases in TFA in students experiencing the art observation activity remotely via Zoom. The pre-post improvement is not different for remote versus in-person participants, and the change in TFA was the same for participants in the museum as well as in remote participation. Further, we observe that age is a significant predictor of TFA change (Table 5). Recent evidence suggests TFA among medical students changes during medical school but in different directions depending on TFA at matriculation [21]. Our results warrant further evaluation to explore the relationship between TFA change and age. At this time, we speculate that age may predict TFA change as a result of maturity level and lived experiences.

We observed minimal or no change in perspective-taking and empathy in our study evaluating participant pre- and post-scores based on the IRI measurement. The lack of significant improvement in perspective-taking and empathy suggests that these traits may be less malleable than tolerance for ambiguity. Alternatively, improvement in perspective-taking and empathy may be observed after multiple museum VTS activities, and the two-hour session was simply not enough time to show results. While neither of these measures showed as large of a change as the change in TFA score, we continue to evaluate if ArtMed course participants' perspective-taking and empathy behaviors and attitudes change after a VTS art observation activity.

While some of the artwork used in our program contained medical themes, the majority of art used during this longitudinal study was selected in order to stimulate dialogue and discussion. The artwork was selected to contain subjects of interest, imagery that represents both familiarity and newness, strong narratives, accessible intrigue, and ambiguity [19]. The VTS process provided the opportunity for students to explore ambiguity and perspective-taking in a psychologically safe space, one that was outside of the competitive environment of the classroom. Introducing topics such as ambiguity in the artwork allowed for a natural transition to ambiguity in patients and in healthcare. The visual arts provided the requisite medium to engage in these conversations.

We have also noted that the visual arts observation activity using VTS provides an opportunity to engage in a discussion of the competencies of interprofessional collaborative practice (IPCP). The small team environment we employed for VTS (10-12 participants), the open dialogue and discussion, and the perspective-taking that occurred during the discussion provide a rich opportunity to discuss and practice the competencies of IPCP. There is also likely broader applicability to address ambiguity, perspective-taking, and empathy across learners of other health professions by structuring the VTS with students of mixed professions.

The COVID-19 pandemic provided the impetus to explore how our arts-based curriculum transitions to an online learning environment. When we had to rapidly transition to remote delivery, we discovered that some of the challenges to creating interprofessional education events and activities (e.g., travel and transportation to the same venue) had eased. Our results show that a remote VTS activity produces comparable outcomes to changes in ambiguity tolerance as the in-museum setting. The change forced upon us due to the COVID-19 pandemic has created the opportunity to expand our visual arts curriculum to include programs across the country. Our live, remote visual arts activity can serve as a model IPE event to bring learners across the health professions together to explore qualities that are essential for strong, collaborative multidisciplinary teams. 

There were a number of limitations in the study. Across three academic years, 672 (85%) students participated in the ArtMed museum activity in an art museum, and 122 (15%) experienced VTS and art observation through Zoom (90 [11%]) during the emergency remote teaching transition and 32 (4%) in planned online remote teaching). In addition, the evolution of the course over three academic years allowed for natural course improvements and improved instructional delivery. Our regression model controls for this as much as possible, but it is important to note course improvement across academic years of instruction. We recognize that holding a discussion with medical students on the subject of ambiguity, empathy, and perspective-taking may influence how participants respond to survey tools that measure these characteristics and traits. Since this was a required course in the curriculum, we do not have an adequate control group and we recognize that some students may not have taken the surveys seriously, potentially skewing the results. 

There is also unknown generalizability of our results to a broader population. While the LMER model suggests that we can expect to observe an increase in tolerance for ambiguity in the theoretical population of medical students from which our sample was randomly drawn after a two-hour art observation activity using VTS, we do not know if these outcomes translate to learners in other health profession programs or practicing health professionals. As described above, this course provides an exciting opportunity for teaching competencies associated with interprofessional collaborative practice. We anticipate evaluating if tolerance for ambiguity, perspective-taking, and empathy changes after an art-based VTS activity in learners from mixed health professions programs as well as mixed groups of healthcare practitioners. 

In addition, there could be other measurements that show a greater impact of this course on medical students. We use three quantitative measurements (tolerance for ambiguity scale, empathy, and perspective-taking), but the VTS-based art observation activity likely has benefits we have not yet been able to measure. We saw a significant change in tolerance for ambiguity scores, but there might also be other measurements that could help us better understand the effect of our required art-based curriculum, such as implicit bias measurements. Future assessment of the course includes evaluating the qualitative outcomes of the course through focus groups and interviews as well as evaluating the sustainability of change with repeat surveys for TFA, perspective-taking, empathy, and other months and years after the museum activity.

## Conclusions

We have developed a curriculum that improves qualities that are essential for practice in complex and evolving health systems. These improvements are observed independent of learning modality (in an art museum, emergency remote teaching, or planned remote teaching), creating an opportunity to engage with students and programs across the spectrum of health professions education, independent of location. Providing access to educational programming that addresses essential qualities and behaviors of healthcare providers has the potential to improve patient outcomes through team-based healthcare and interprofessional collaborative practice.
